# Rapid Identification of Medically Important *Candida* Isolates Using High Resolution Melting Analysis

**DOI:** 10.1371/journal.pone.0116940

**Published:** 2015-02-17

**Authors:** Eva Nemcova, Michaela Cernochova, Filip Ruzicka, Barbora Malisova, Tomas Freiberger, Petr Nemec

**Affiliations:** 1 Centre for Cardiovascular Surgery and Transplantation Brno, Brno, Czech Republic; 2 International Clinical Research Centre, St. Anne's University Hospital Brno, Brno, Czech Republic; 3 Molecular Immunology and Microbiology, Central European Institute of Technology, Masaryk University, Brno, Czech Republic; 4 Department of Microbiology, Faculty of Medicine, Masaryk University and St. Anne's University Hospital Brno, Brno, Czech Republic; Institute of Microbiology, SWITZERLAND

## Abstract

An increasing trend in non *albicans* infections and various susceptibility patterns to antifungal agents implies a requirement for the quick and reliable identification of a number of medically important *Candida* species. Real-time PCR followed by high resolution melting analysis (HRMA) was developed, tested on 25 reference *Candida* collection strains and validated on an additional 143 clinical isolates in this study. All reference strains and clinical isolates inconclusive when using phenotypic methods and/or HRMA were analysed using *ITS2* sequencing. Considering reference and clinical strains together, 23 out of 27 *Candida* species could be clearly distinguished by HRMA, while the remaining 4 species were grouped in 2 pairs, when applying the mean Tm ± 3 SD values, the shape of the derivative melting curve (dMelt curve) and, in some cases, the normalized and temperature—shifted difference plot against *C. krusei*. HRMA as a simple, rapid and inexpensive tool was shown to be useful in identifying a wide spectrum of clinically important *Candida* species. It may complement the current clinical diagnostic approach based on commercially available biochemical kits.

## Introduction

Candidiasis is a dominant fungal infection worldwide and candidemia represents the fourth most common bloodstream infection in hospitalized patients in the United States [1,2] and the seventh in Europe [3,4]. Although *Candida albicans* remains the most common fungal isolate from blood, many studies have reported an increasing trend in non-*albicans* infections [5–9]. Horn *et al*. (2009) [10] described an even higher incidence of candidemia caused by non-*albicans Candida* species than *C*. *albicans* in the United States.

The correct and quick identification of *Candida* species is very important, particularly for providing adequate antifungal therapy based on species-dependent susceptibility patterns [11], but also for epidemiological purposes [12]. Candidiasis diagnosis is based on microscopy, histopathology, culture and chromogenic culture media, as well as enzyme, metabolite, *Candida* cell wall structural component (1,3-β-D-glucan) and antibody (antimannan) or antigen (mannan) detection [12,13].

Molecular techniques are not recommended for diagnosing candidiasis in clinical practise because of insufficient data and lack of standardization [13]. However, many applications relying mainly on PCR have been developed to detect candidal DNA and may be useful in specific situations. Further species identification was carried out using restriction fragment length polymorphism (RFLP) [14,15], sequencing [16–18], pyrosequencing [19–21], real-time PCR with specific probes [22–26] or real-time PCR with species specific or pan-fungal primers [27–29]. Other authors applied semi-nested PCR [30], multiplex tandem PCR [31], single-strand conformation polymorphism (SSCP) [32], peptide nucleic acid fluorescent in situ hybridization (PNA FISH) [33], matrix-assisted laser desorption/ionization (MALDI-TOF) [34–36], nucleic acid sequence based amplification (NASBA) [37] or PCR coupled to electrospray ionization mass spectrometry (PCR/ESI-MS) [38]. Unfortunately, these approaches are either expensive, laborious, not proven to differentiate multiple *Candida* species simultaneously or lack verified comprehensive databases containing uncommon/emerging species.

Another strategy is based on high resolution melting analysis (HRMA), which can be used for species identification although it was developed originally for genotyping, mutation scanning and sequence matching [39]. This method has already been applied in bacteriology [40–43], virology [44,45] and mycology [46–54]. In this study we report a method combining broad-range real-time PCR and HRMA for the rapid identification of 25 medically important *Candida* species from culture, verified on clinical isolates.

## Material and Methods

### Fungal strains

Twenty five medically important *Candida* reference strains (Table 1) were selected from the Belgian Co-ordinated Collections of Micro-organisms (BCCM/MUCL), the Czech Collection of Microorganisms (CCM) and the Culture Collection of Yeasts (CCY): *C*. *albicans* (CCM 8320), *C*. *catenulata* (CCY 29-17-3), *C*. *dubliniensis* (CCY 29-177-1), *C*. *fabianii* (CCY 38-20-1), *C*. *famata* (CCY 26-9-9), *C*. *glabrata* (CCM 8270), *C*. *guilliermondii* (CCY 39-23-6), *C*. *inconspicua* (CCY 26-26-11), *C*. *intermedia* (CCY 29-12-10), *Kluyveromyces marxianus* (*C*. *kefyr)* (MUCL 29857), *C*. *krusei* (CCY 29-9-17), *C*. *lambica* (CCY 29-97-12), *C*. *lipolytica* (CCY 29-26-42), *C*. *lusitaniae* (CCY 29-59-1), *C*. *metapsilosis* (MUCL 46179), *C*. *norvegensis* (CCY 29-47-2), *C*. *orthopsilosis* (MUCL 49939), *C*. *parapsilosis* (CCM 8260), *C*. *pelliculosa* (CCY 29-6-7), *C*. *pulcherrima* (CCY 29-2-128), *C*. *rugosa* (CCY 29-15-1), *C*. *tropicalis* (CCM 8264), *C*. *utilis* (CCY 29-38-74), *C*. *valida* (CCY 29-93-4), *C*. *zeylanoides* (MUCL 27735) for the analysis.

**Table 1:** Reference strains’ Tm and *ITS2* sequence characteristics.

species	reference strain No.	**mean Tm (SD)°C** ^a^		Length (bp)	GC content (%)	**GenBank Acc. No.** ^b^	**CBS No.** ^b^
		melting peak 1	melting peak 2				
C. albicans	CCM 8320	80.098 (0.048)	82.938 (0.085)	326	47	FJ662402.1	CBS 8758
C. californica	CCY 29-93-4	85.170 (0.083)	-	288	53	AY684146.1	CBS 989
C. catenulata	CCY 29-17-3	79.730 (0.067)	82.850 (0.028)	259	46	AY493436.1	CBS 565
C. dubliniensis	CCY 29-177-1	80.200 (0.064)	82.090 (0.089)	331	46	HQ457429.1	CBS 7987
C. fabianii	CCY 38-20-1	81.083 (0.051)	-	361	44	AF335967.1	CBS 5640
C. glabrata	CCM 8270	81.705 (0.070)	-	408	46	AY939793.1	CBS 859
C. guilliermondii	CCY 39-23-6	81.255 (0.039)	-	368	44	JQ425356.1	CBS 6021
C. inconspicua	CCY 26-26-11	84.603 (0.112)	-	295	52	AB179767.1	CBS 180
C. intermedia	CCY 29-12-10	81.968 (0.032)	-	247	47	AF218968.1	CBS 572
C. kefyr	MUCL 29857	82.658 (0.084)	-	422	46	HQ396523.1	CBS 834
C. krusei	CCY 29-9-17	86.605 (0.043)	-	336	55	L47113.1	CBS 2062
C. lambica	CCY 29-97-12	87.678 (0.039)	-	295	58	AY533549.1	CBS 4807
C. lipolytica	CCY 29-26-42	78.325 (0.139)	81.710 (0.052)	229	44	HQ718589.1	CBS 5699
C. lusitaniae	CCY 29-59-1	83.663 (0.079)	-	244	50	EU149777.1	CBS 1944
C. metapsilosis	MUCL 46179	79.333 (0.093)	81.213 (0.083)	304	42	FJ872019.1	CBS 10747
C. norvegensis	CCY 29-47-2	86.930 (0.087)	-	314	54	AB179768.1	CBS 1922
C. orthopsilosis	MUCL 49939	79.563 (0.037)	81.208 (0.034)	300	43	EU564208.1	CBS 10745
C. parapsilosis	CCM 8260	79.863 (0.071)	81.508 (0.061)	300	43	HQ263346.1	CBS 2211
C. pelliculosa	CCY 29-6-7	78.625 (0.060)	79.683 (0.065)	364	39	AF218991.1	CBS 605
C. pulcherrima	CCY 29-2-128	82.178 (0.062)	-	243	48	FJ172526.1	CBS 9701
C. rugosa	CCY 29-15-1	83.980 (0.079)	-	263	49	GU144663.1	CBS 613
C. saitoana	CCY 26-9-9	79.695 (0.052)	-	361	41	HQ652067.1	CBS 940
C. tropicalis	CCM 8264	79.433 (0.061)	80.738 (0.096)	317	40	EU589208. 1	CBS 94
C. utilis	CCY 29-38-74	80.720 (0.073)	-	352	45	AF218990.1	CBS 567
C. zeylanoides	MUCL 27735	79.328 (0.063)	-	363	40	AF218976.1	CBS 6409

^a^Tm–melting temperature, SD–standard deviation–calculated using Tm values of reference strains analysed in duplicates in 3 independent runs

^b^ homologous *ITS2* sequence in database

In addition, 143 available *Candida* isolates phenotypically corresponding to selected reference strains, cultured from different clinical specimens, were included in the study.

### DNA extraction

Pure cultures of all *Candida* reference strains and clinical isolates were homogenized in 100 μl of sterile water (B. Braun Medical, Inc., Germany) and incubated with lysis buffer (EDTA, Tris, Triton X-100), lysozyme (Sigma-Aldrich, USA) and lyticase (Sigma-Aldrich, USA) for 60 min at 37°C to disrupt the fungal cell wall. The DNA was isolated by using the fungal DNA isolation protocol with QIAamp DNA Blood Mini Kit (Qiagen, Germany). DNA purity and concentrations were measured using NanoDrop ND-1000 spectrophotometer (NanoDrop Technologies, Inc., North Carolina, USA).

### Confirmation of strains

Extracted fungal DNA was used for PCR targeting the *ITS2* region (primers UNF1 and UNF2 published previously [14] and run under the following conditions: 10 min at 96°C, then 45 cycles of 10 s at 98°C, 10 s at 56°C, 30 s at 72°C and a final step of 4 min at 72°C. Purified amplicons were subjected to cycle sequencing using a Big Dye Terminator 3.1 Cycle Sequencing Kit (Life Technologies, California, USA) and sequenced on ABI Prism 3100 Avant (Life Technologies). The sequences were put to alignment analysis using the BLAST tool of the National Centre for Biotechnology Information (NCBI) (http://www.ncbi.nlm.nih.gov/blast, accessed Dec 2013) [55] and to the pairwise sequence alignment tool of the Fungal Biodiversity Centre database (http://www.cbs.knaw.nl/Collections/BioloMICSSequences.aspx?file=all, accessed Dec 2013) [56] (Table 1).

All clinical isolates were tested using conventional biochemical methods, API-ID32C (BioMérieux, France) and CANDIDAtest 21 (Erba-Lachema, Czech Republic). *C*. *dubliniensis* was identified using the Bichro-Dubli Fumouze additional latex agglutination test (Fumouze Diagnostics, France). Clinical isolate sequencing was only carried out in cases when the HRMA pattern did not correspond with any reference strain, some inconsistencies in HRMA pattern were noted, phenotypic and HRMA testing differed in species determination or the phenotype was not determined unambiguously. MEGA4 software [57] was used for *C*. *metapsilosis* reference and clinical strain sequence alignment. Reference strains GC content (%) was determined using uMELT software v2.0.2 [58] (Table 1).

### PCR conditions and HRMA

Real-time PCR amplification and HRMA of the *ITS2* region were performed on the Rotor-Gene 6000 (Corbett Research, Austria) with primers UNF1 and UNF2, the same as were used for sequencing. PCR was carried out with 1 × SensiMix HRM (Quantace, United Kingdom), 20 ng of fungal DNA, 3.5 mmol/l MgCl_2_ (Fermentas UAB, Lithuania), 1.5 × saturating dye EvaGreen (Biotium, USA), 0.35 μmol/l of each primer (final concentration) and sterile water up to 25 μl (B. Braun Medical, Germany). The PCR program consisted of an initial denaturation of 10 min at 95°C, followed by 40 cycles of 10 s at 95°C, 15 s at 58°C and 30 s at 72°C, and ended with 4 min at 72°C. The amplification products for HRMA (229–422 bp long with GC content 39–58%) were cooled 1 min at 50°C and then heated from 70°C to 98°C monitoring fluorescence at the rate of 0.15°C/2 s. Melting data were normalised and the temperature shifted using Rotor-Gene 6000 analysis software version 1.7 (Corbett Research, Austria). Normalisation regions for HRMA were set at 71–73°C and 95–97°C. All reference strains were analysed in duplicates in 3 independent runs and the mean Tm and SD values were calculated for each melting domain. Different Tm value ranges were tested and finally the mean Tm ± 3 SD range was selected to overcome the interassay variability and reach maximum sensitivity and reproducibility. Then, randomly determined numbers of isolates (21 to 33) were tested per each HRMA run. Each clinical strain was evaluated by comparing its Tm value(s) with established reference intervals of mean Tm ± 3 SD and by simple visual inspection of its derivative melting curve shape (dMelt curve shape). If this approach did not result in unambiguous identification, a difference plot of analysed species against *C*. *krusei* was performed. Three positive controls were used in each assay (*C*. *krusei*, *C*. *norvegensis*, *C*. *saitoana*).

## Results

### Sequencing of reference strains

All reference strains except CCY 26-9-9 and CCY 29-93-4 were validated as those supplied by culture collections by *ITS2* sequencing and alignment analysis (Table 1). Reference strains signed as *C*. *famata* (CCY 26-9-9) and *C*. *valida* (CCY 29-93-4) were re-identified as *C*. *saitoana* and *C*. *californica* respectively, using *ITS2* sequencing.

### HRMA in reference strains

**Figure 1:**
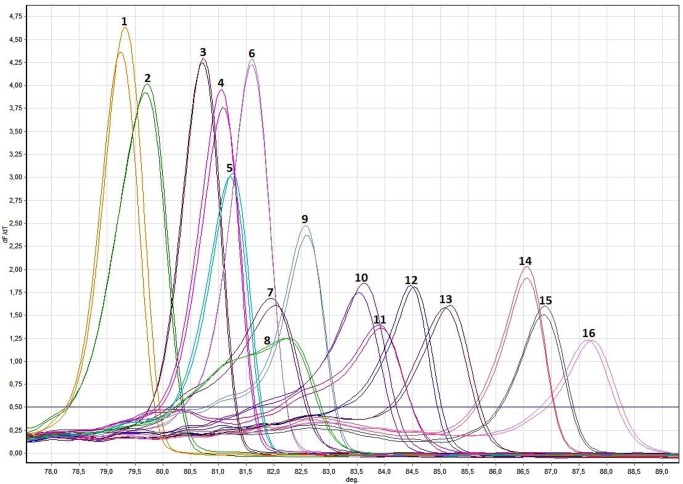
dMelt curves of reference *Candida* sp. in duplicates in *Candida* strains with a single Tm value. 1) *C*. *zeylanoides*, 2) *C*. *saitoana*, 3) *C*. *utilis*, 4) *C*. *fabianii*, 5) *C*. *guilliermondii*, 6) *C*. *glabrata*, 7) *C*. *intermedia*, 8) *C*. *pulcherrima*, 9) *C*. *kefyr*, 10) *C*. *lusitaniae*, 11) *C*. *rugosa*, 12) *C*. *inconspicua*, 13) *C*. *californica*, 14) *C*. *krusei*, 15) *C*. *norvegensis*, 16) *C*. *lambica*

**Figure 2:**
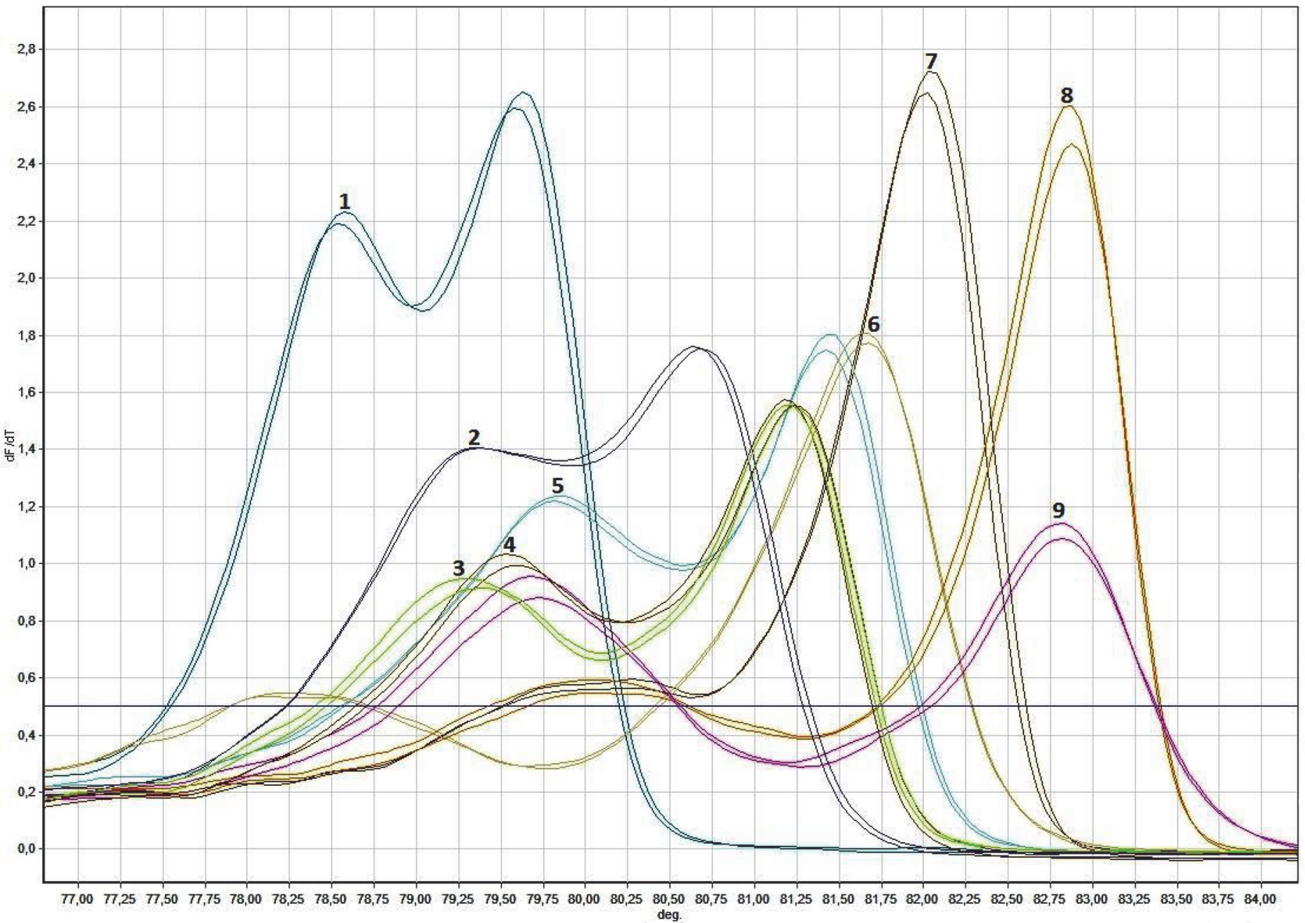
dMelt curves of reference *Candida* sp. in duplicates in *Candida* strains with two Tm values. Scale different from Fig. 1: 1) *C*. *pelliculosa*, 2) *C*. *tropicalis*, 3) *C*. *metapsilosis*, 4) *C*. *orthopsilosis*, 5) *C*. *parapsilosis*, 6) *C*. *lipolytica*, 7) *C*. *dubliniensis*, 8) *C*. *albicans*, 9) *C*. *catenulata*

Real-time PCR followed by HRMA, performed with 25 *Candida* reference strains, showed reproducible melting peaks for each species. Most of them displayed a single peak, whereas nine *Candida* species melted in two domains (Table 1, Figs. 1, 2).

**Figure 3:**
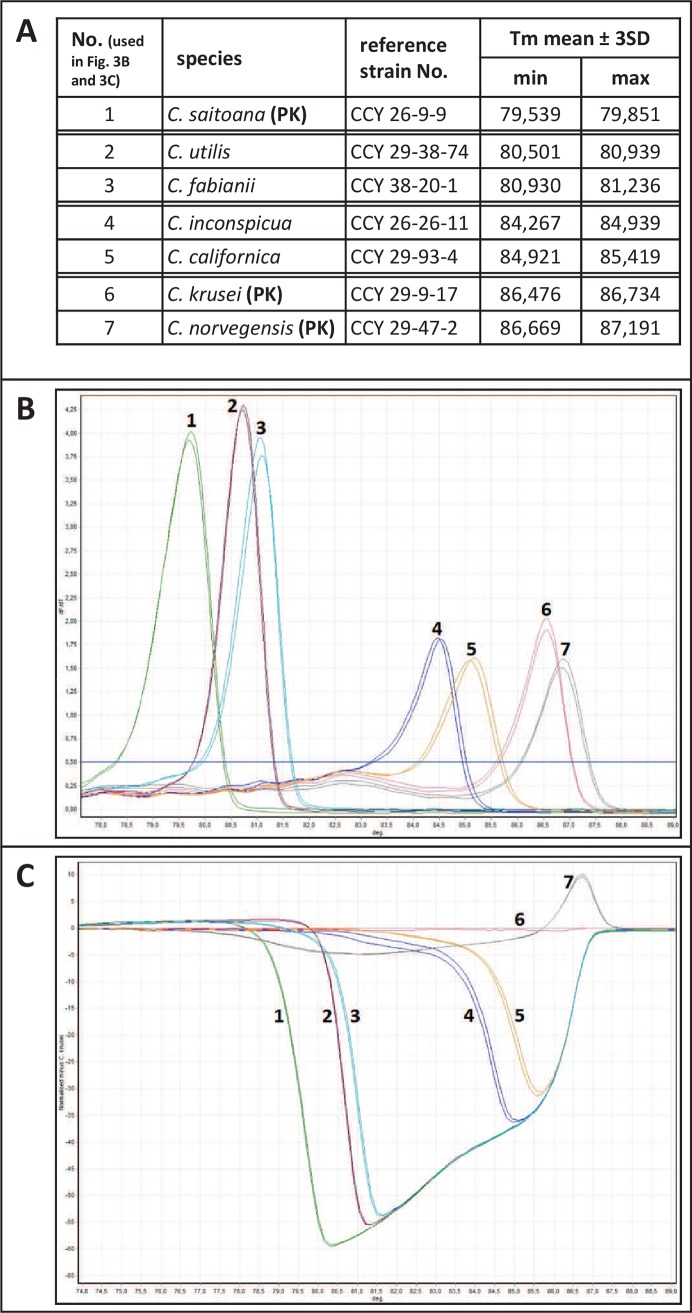
HRMA identification procedure in species with overlapping Tm ranges. **A)** Tm ± 3 SD reference strains values, **B)** shapes of the selected reference strains dMelt curves, **C)** normalized and temperature–shifted difference plot of the reference strains against *C*. *krusei*. PK–positive control used in each HRMA run.

Unique patterns were identified in 21 out of 25 *Candida* species directly after melting curve analysis, including *C*. *saitoana* and *C*. *californica* instead of *C*. *famata* and *C*. *valida*, respectively. The mean Tm ± 3 SD value(s) together with the dMelt curve shape were conclusive in 16 species, while normalized temperature-shifted difference plot against *C*. *krusei* had to be used to distinguish between two species in the other 5 reference strains (three couples), *C*. *inconspicua* vs. *C*. *californica*, *C*. *krusei* vs. *C*. *norvegensis*, and *C*. *utilis* vs. *C*. *fabianii* (Fig. 3), the latter, however, could not be distinguished from *C*. *guilermondii* (see below).

Four species (two pairs) could not be reliably distinguished. Tm ± 3SD values for *C*. *orthopsilosis* and *C*. *metapsilosis* occurred repeatedly in clearly overlapping ranges, and similarly, the reference strains of *C*. *fabianii* and *C*. *guilliermondii*, despite very different sequences, had significantly overlapping margins of Tm ± 3SD ranges. Neither the curve shape nor plotting had any additional value in these cases. Nevertheless, both *C*. *orthopsilosis* and *C*. *metapsilosis* were clearly distinguishable from *C*. *parapsilosis*.

The sensitivity of the assay was determined by serially diluted *C*. *albicans* reference strain DNA in duplicates. Concentrations of 50 ng–1 pg provided reliable HRMA patterns.

### HRMA in clinical isolates

All clinical isolates (n = 143) were tested in a blinded manner. When the HRMA pattern of a particular clinical isolate was different from the melting data in a reference panel, the isolate was sequenced to clarify an observed discrepancy. Eleven samples showing mixed electropherogram were excluded from the primary analysis. An additional seven clinical isolates representing 2 species could not be identified, because they were not included in the reference list (three *C*. *pararugosa* and four *C*. *famata* samples) (Table 2, bottom). However, all of these 7 samples revealed a reliable pattern, either directly (*C*. *pararugosa*) or after being plotted against *C*. *krusei* (*C*. *famata*), and could be differentiated from every other species. Thus, 125 clinical isolates (21 species) finally corresponded to the set of reference strains (Table 2, top).

**Table 2:** *Candida* clinical isolates identified by phenotypic methods, HRMA and sequencing.

		HRMA			Phenotypic methods		
	Clinical isolates (n)^a^	Correct identification^b^	Correct species group ranging^c^	Not identified correctly^c^	Correct identification	Correct species group ranging^c^	Not identified correctly^c^
reference strains							
C. albicans	13	9 + 4^d^	-	-	13	-	-
C. catenulata	1	1	-	-	1	-	-
C. dubliniensis	5	5	-	-	5^h^	-	-
C. fabianii	20	-	20^e^	-	-	-	20^g^
C. glabrata	10	10	-	-	10	-	-
C. guilliermondii	6	-	6^e^	-	6	-	-
C. inconspicua	1	1	-	-	1	-	-
C. intermedia	1	1	-	-	1	-	-
C. kefyr	6	6	-	-	6	-	-
C. krusei	7	7	-	-	7	-	-
C. lambica	3	-	-	3^d^	3	-	-
C. lipolytica	3	3	-	-	3	-	-
C. lusitaniae	5	5	-	-	5	-	-
C. metapsilosis	9	-	3^f^ + 4^f^ ^,^ ^d^	2^d^	-	-	9^i^
C. norvegensis	4	4	-	-	4	-	-
C. orthopsilosis	5	-	5^f^	-	-	-	5^i^
C. parapsilosis	15	15	-	-	15	-	-
C. pelliculosa	3	3	-	-	-	3^g^	-
C. pulcherrima	1	1	-	-	1	-	-
C. tropicalis	6	6	-	-	6	-	-
C. utilis	1	1	-	-	-	1^g^	-
Subtotal: isolates	125 (100%)	82 (65.6%)	38 (30.4%)	5 (4.0%)	87 (69.6%)	4 (3.2%)	34 (27.2%)
Subtotal: species	21 (100%)	16 (76.2%)^j^	4 (19.0%)^j^	2 (9.5%)^j^	16 (76.2%)	2 (9.5%)	3 (14.3%)
out of reference strains							
C. famata	4	4	-	-	4	-	-
C. pararugosa	3	3	-	-	-	-	3
Total: isolates	132 (100%)	89 (28.8%)	38 (28.8%)	5 (3.8%)	91 (69.0%)	4 (3.0%)	37 (28.0%)
Total: species	23 (100%)	18 (78.3%)^j^	4 (17.4%)^j^	2 (8.7%)^j^	17 (73.9%)	2 (8.7%)	4 (17.4%)

^a^poly-fungal samples were excluded

^b^using Tm ± 3SD range (shown in the Table 1) and dMelt curve shape

^c^all samples were verified by sequencing

^d^heterogeneities were identified (see the text for details)

^e^not possible to distinguish between *C*. *guilliermondii/C*. *fabianii*

^f^not possible to distinguish between *C*. *orthopsilosis/C*. *metapsilosis*

^g^identified as *C*. *utilis/C*. *pelliculosa*

^h^reliably distinguished from *C*. *albicans* using aditional Bichro-Dubli Fumouze latex-aglutination test

^i^identified as *C*. *parapsilosis*

^j^total percentage exceeds 100% due to seven isolates *C*. *metapsilosis* in “correct group ranging” and two isolates in “not identified” category.

Eighty-two of them (65.6%), representing 16 species (76.2%), were identified correctly to species level using either Tm ± 3 SD ranges calculated from the corresponding reference strains only or in connection with the dMelt curve shape. Despite overlapping Tm ± 3 SD ranges in the reference strains in pairs of *C*. *utilis/C*. *fabianii*, *C*. *inconspicua/C*. *californica* and *C*. *krusei*/*C*. *norvegensis*, no plotting against *C*. *krusei* was needed in clinical isolates of these species, because their Tm values did not fall into these overlapping areas. Sequencing revealed some species heterogeneities in *C*. *albicans* (differed by 1 bp from the reference sequence in 4/13 isolates), *C*. *norvegensis* (3 bp in 4/4), *C*. *intermedia* (1 bp in 1/1) and *C*. *lipolytica* (1 bp in 1/3). However, the correct species identification was affected in none of these cases.

The other 38 isolates (30.4%), representing 4 species (19.0%), were ranged properly, but could not decide between two species (*C*. *fabianii*/*C*. *guilliermondii* in 26 and *C*. *orthopsilosis*/*C*. *metapsilosis* in 12 cases). Sequence variants in 1 bp detected by sequencing in 6/6 *C*. *guillermondii* and 4/9 *C*. *metapsilosis*, compared to the corresponding reference strains, did not affect the result.

**Figure 4:**
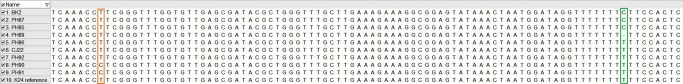
Partial sequence of nine *C*. *metapsilosis* clinical isolates and reference strain. (amplicon position 141 bp and 218 bp highlighted)

Five clinical isolates (4.0%), belonging to the two species (9.5%), could not be identified at all (*C*. *lambica* in 3/3 and *C*. *metapsilosis* in 2/9 cases). In these isolates, variations in 4 bp and 1 bp respectively, compared to reference strains, were demonstrated, which shifted the Tm value out of the Tm ± 3 SD range calculated for the respective reference strains and identification failed. All of these 5 strains showed a unique pattern and could not be misidentified with any other clinical or reference strain included in our panel. Thus, two different heterogeneities differing by 1 bp from the reference strain were identified in *C*. *metapsilosis* clinical isolates (Fig. 4). While a difference in position 218 of the amplicon did not affect the Tm and the dMelt curve shape significantly, another difference in position 141 resulted in three-peak pattern and disabled the identification.

In 2 out of 11 samples showing poly-fungal pattern on electropherogram, species identification was not possible at all. However, in the remaining 9 isolates the dMelt curve showed only a small deviation from the respective reference curve and the major species could be identified correctly and corresponded to phenotypic identification. Notably, the phenotypic approach failed to identify a minor strain in all these cases.

### Phenotypic methods in clinical isolates

Based on phenotypic methods, all 5 *C*. *orthopsilosis* and 9 *C*. *metapsilosis* isolates were misidentified as *C*. *parapsilosis*, and 20 *C*. *fabianii* isolates were misidentified as *C*. *utilis*/*C*. *pelliculosa*. In an additional 4 isolates, *C*. *pelliculosa* and *C*. *utilis* could not be distinguished from each other. Three clinical isolates delivered to our lab as *C*. *rugosa* were finally determined using sequencing as *C*. *pararugosa*. All other clinical strains were identified correctly (Table 2).

## Discussion

In this study, we combined traditional culture with real-time PCR and HRMA for rapid identification of *Candida* pathogens. A newly developed HRMA was optimized and tested on 25 reference collection strains, of which 21 were distinguished unambiguously. Using this method, 143 phenotypically defined clinical isolates were tested in a blinded manner and melting data, particularly the mean Tm ± 3 SD and the dMelt curve shape, were compared to the reference panel. Evaluating all clinical isolates, the samples were identified correctly in 18 out of 23 species supplied (Table 2). In addition, the clinical isolates of another species (*C*. *lambica*) were distinguished, although the identification failed due to sequence differences from the reference strain. All four species unavailable as clinical isolates, *C*. *californica*, *C*. *saitoana*, *C*. *rugosa* and *C*. *zeylanoides*, were clearly distinguishable when applying for collection strains.

Considering the reference and clinical strains together, 23 out of 27 *Candida* species were distinguished, while the remaining 4 species were grouped in 2 pairs, using HRMA in this study. To our knowledge, this is the widest spectrum of *Candida* species tested and distinguished using the HRMA method so far, with the large number of 143 clinical isolates covering 23 species used for validation. Three *Candida* species were used as positive controls in each run. Mandviwala *et al*. (2010) [49] differentiated 8 *Candida* species based on *ITS1* and *ITS2* sequences, when using a relatively demanding analysis in MS Excel. Arancia *et al*. (2011) [50] developed a procedure to identify 5 *Candida* sp. using *MP65* gene with analysis made only following normalized and derivated melting curve shapes. Goldschmidt *et al*. (2012) [52] reported the HRMA approach based on *18S rDNA* sequence to distinguish 5 *Candida* sp. simultaneously with 6 filamentous fungi, which were however necessary for further analysis (plotting) and the identification of *Candida* sp. Somogyvari *et al*. (2012) [51] differentiated 10 *Candida* sp. analysing the HRMA of the *ITS2* sequence, but all expected species had to be used as positive controls in each run to get reliable results. Decat *et al*. (2013) [53] described an approach enabling the identification of 17 *Candida* sp. and one other pair based on the *ITS2* sequence, using 5 positive controls in each run and two plotting schemes to interpret data. Alnuaimi *et al*. (2014) [54] used HRMA to distinguish 9 *Candida* sp. based on *ITS1*, *5.8S* and *ITS2* sequences, having 8 species as positive controls in each run. Despite HRMA performing well in this study, unequivocal identification could not be carried out in 4 reference strains (*C*. *orthopsilosis* vs. *C*. *metapsilosis*, *C*. *fabianii* vs. *C*. *guillermondii*) and 5 clinical isolates (*C*. *orthopsilosis* vs. *C*. *metapsilosis*, *C*. *fabianii* vs. *C*. *guillermondii*, *C*. *lambica*). Tm ± 3SD values for both reference and clinical isolates of *C*. *orthopsilosis* and *C*. *metapsilosis* occurred repeatedly in overlapping ranges, therefore these two species were subsumed into one undistinguishable group. However, routine discrimination within this group is not considered to be necessary and even commercial biochemical tests do not differentiate among species in the *C*. *parapsilosis* complex [59]. On the other hand, distinguishing *C*. *orthopsilosis*/*C*. *metapsilosis* from *C*. *parapsilosis* might be of importance, as variations in antifungal susceptibility with *C*. *orthopsilosis* and *C*. *metapsilosis* being more susceptible to antifungals than *C*. *parapsilosis* have been described [60]. Notably, all *C*. *parapsilosis* clinical isolates representing the medically most important organism in the *psilosis group were clearly distinguishable from *C*. *orthopsilosis*/*C*. *metapsilosis* strains using HRMA.

Interestingly, *C*. *metapsilosis* clinical isolates revealed three different types of heterogeneities (Fig. 4), two variants did not affect the correct species group ranging, but the other one caused three melting peaks instead of two in the Tm curve shape which prevented correct identification, although the HRMA pattern could not be confused with the curve of any other species tested. Similarly, the HRMA data of all 3 clinical isolates of *C*. *lambica* failed to match with the corresponding reference strain pattern, as they all differed by the same 4 nucleotides. Although the identification of clinical samples of *C*. *lambica*, could not be completed, all of them showed a unique curve avoiding confusion with any other species. HRMA is a sensitive method capable of discriminating sequences with minimal variations, which on the other hand can hamper correct pathogen identification at species level in cases of intraspecies variability. Nevertheless, this limitation may even become an advantage when using HRMA in typing clinical isolates for epidemiological reasons [54].

Despite very different sequences of *C*. *fabianii* and *C*. *guilliermondii*, HRMA could not differentiate between them, as their Tm ± 3SD ranges significantly overlapped, most likely due to the identical GC content both in reference and clinical isolate sequences. Using phenotypic determination, *C*. *guilliermondii* can be recognized unambiguously between these two species. Although not occurring in our clinical samples, biochemical identification can misidentify *C*. *guilliermondii* as *C*. *famata* (*D*. *hansenii*) [61] which, on the contrary, can reliably be distinguished using HRMA (as we demonstrated on clinical strains). Thus, combining both approaches all three species could be decidedly identified. In addition, the phenotypic differentiation of *C*. *fabianii*, *C*. *pelliculosa* and *C*. *utilis* is not easy since all have a similar pattern and could be mutually misidentified [62], which ultimately happened in our clinical samples. *C*. *fabianii* is included neither in CandidaTest 21, nor in API-ID32C although it is able to form biofilm and cause severe infections [62,63]. All *C*. *pelliculosa*, *C*. *utilis* and *C*. *fabianii* isolates were convincingly distinguishable using HRMA in our testing.

*C*. *albicans* and *C*. *dubliniensis* differentiation is usually based on biochemical tests exhibiting the ability of *C*. *dubliniensis* not to assimilate *D-xylosis* (XYL), *methyl-α-D-glucoside* (MDG) and *D-trehalose* (TRE), but neither these tests nor tests based on pseudohyphal and hyphal formation on special culture media, e.g. Staib agar [64] and chlamydospore production give unambiguous results [65,66]. The additional use of the Bichro-Dubli Fumouze latex-agglutination test enables reliable identification [67], as we demonstrated in our set of clinical isolates, and similarly HRMA had characteristic dMelt curves for both species.

Sequencing showed poly-fungal pattern with a prevalent sequence corresponding to the species identified by HRMA in nine clinical isolates, while phenotypic analysis reported them as a single species. A minority strain in these poly-fungal samples slightly affected the shape of the dMelt curve but did not compromise the identification of the prevailing strain. HRMA also detected two poly-fungal isolates, which could not be specified.

Several of HRMA’s other superiorities over routinely used *Candida* identification methods were shown in our study. Unlike HRMA, API-ID32C (including 34 *Candida* species except *C*. *pararugosa*) is not able to distinguish *C*. *norvegensis* and *C*. *inconspicua*, CANDIDAtest 21 does not include either *C*. *pulcherrima* or *C*. *zeylanoides* among 24 *Candida* species and, moreover, it is not able to differentiate *C*. *rugosa* and *C*. *pararugosa*. Even pyrosequencing failed to identify *C*. *pararugosa*, *C*. *lambica* and *C*. *lipolytica* [19,20] which could be clearly distinguished using HRMA.

Real-time PCR followed by HRMA is a simple, efficient and rapid method of identifying cultured *Candida* sp. our time to result after culture positivity was 6 hours compared to 24–48 hours required for the biochemical methods used in this study. This approach might be useful even to identify *Candida* sp. directly from biological samples, as has already been demonstrated by others [49,50,52]. Applying our HRMA directly to clinical samples would lead to further reducing the time to result, but additional studies are needed to validate this method for such a setting. However, this assay can be useful to complement current clinical diagnostic methods for *Candida* isolates, mainly when phenotypically similar *Candida* species or those not included in the biochemical tests are suspected.
